# Radiation dose and shielding considerations for digital dynamic radiography (DDR) compared to mobile C‐arms

**DOI:** 10.1002/acm2.70256

**Published:** 2025-09-03

**Authors:** Azmul Siddique, Gary Ge, Jie Zhang

**Affiliations:** ^1^ Department of Radiology University of Kentucky Lexington Kentucky USA

**Keywords:** DDR, digital dynamic radiography, dose, dose rate, shielding

## Abstract

**Background:**

Digital dynamic radiography (DDR), integrated into Konica Minolta's portable mKDR system, provides dynamic imaging for pulmonary, orthopedic, and interventional applications. While DDR is not classified as fluoroscopy, its use of pulsed x‐rays for cine‐like image sequences raises concerns about radiation exposure and shielding, particularly given the absence of a primary beam stop, high output capabilities, and increasing clinical adoption.

**Purpose:**

To characterize the primary and scatter radiation output of a DDR system and compare it against commonly used mobile C‐arm fluoroscopy units, and to evaluate shielding requirements and potential occupational exposure risks associated with DDR use.

**Methods:**

Radiation dose output and scatter were assessed for a Konica Minolta mKDR system and three mobile C‐arms: GE OEC Elite, Siemens Cios Spin, and Ziehm Vision RFD 3D. Unshielded primary air kerma was measured at 100 cm SID using matched dose settings (low, medium, high). Scatter fraction and normalized scatter were measured at eight angles and three distances using a 20 cm PMMA phantom and an ion chamber. Additional direct comparisons of angular scatter doses between DDR and a GE C‐arm were made during 20‐s acquisitions at varying distances. The Klein–Nishina differential cross section was also calculated for photon energies representative of clinical settings. Leakage radiation and image receptor attenuation were quantified. Shielding requirements were estimated using NCRP 147 methodology under varying workload and occupancy conditions.

**Results:**

DDR exhibited dose rates two to three times higher than C‐arms at medium and high dose settings, with longer pulse widths (16 ms) producing greater exposure than shorter ones (5 ms). Scatter fraction peaked at 165° and increased with lower beam energy due to energy‐dependent Compton interactions and reduced filtration. Compared to the GE C‐arm, DDR produced consistently higher scatter values at all angular positions. Measured scatter doses at 0.3 m and 1.0 m from the phantom exceeded those from the C‐arm, especially in the forward direction (0°). Image receptor attenuation measurements showed 98% beam reduction when the receptor was properly aligned. Leakage was minimal and well below FDA limits. Shielding assessments indicated that concrete thickness requirements for DDR could reach 145 mm under worst‐case conditions, driven primarily by the high primary beam output rather than scatter or leakage.

**Conclusions:**

DDR systems provide portable dynamic imaging capabilities but deliver substantially higher radiation output than conventional mobile C‐arms. In addition, scatter dose rates from DDR were approximately 1.5–3 times higher than those from a conventional mobile C‐arm under comparable conditions. This elevated dose, driven by high tube currents and long pulse durations, raises important safety concerns for patients, personnel, and shielding infrastructure. While DDR offers potential clinical value in motion‐sensitive applications, its safe integration into practice requires careful protocol selection, attention to scatter exposure, and thoughtful shielding planning of exam rooms where the system will be used. As DDR systems become more prevalent and approach fluoroscopic performance, regulatory and design guidance may need to evolve to reflect their unique operational profile.

## 1 INTRODUCTION

New imaging technologies continue to emerge, offering potential benefits for various clinical procedures and applications. It is essential to evaluate these applications carefully, particularly in terms of radiation safety for both patients and healthcare personnel. One such technology is Konica Minolta's Digital Dynamic Radiography (DDR). Since its commercial debut in 2022 for portable systems, DDR has seen increasing adoption in clinical practice.

DDR is integrated into Konica Minolta's portable radiographic system and provides dynamic imaging that facilitates many clinical cases such as evaluating pulmonary circulation, mainly in patients with chronic thromboembolic pulmonary hypertension (CTEPH), offering significant advantages such as reduced radiation exposure compared to traditional scintigraphy.^1^ In orthopedics, DDR enhances the diagnosis of posterior shoulder instability by capturing dynamic joint movements, thereby providing more detailed insights than static imaging methods.^2^ Another example is dynamic perfusion digital radiography (DPDR) that plays a crucial role in predicting postoperative pulmonary function after lung cancer resections. Its predictive accuracy closely aligns with that of established techniques, that is lung perfusion scan, while being more cost‐effective and easier to implement.^3^ In the context of chronic obstructive pulmonary disease (COPD), dynamic‐ventilatory digital radiography (DVDR) offers valuable information about lung function and treatment efficacy, positioning itself as a non‐invasive alternative for patient assessment.^4^ This DDR system can also be utilized in cases requiring life support, such as extracorporeal membrane oxygenation (ECMO) cannulation procedures, where it aids in verifying cannula placement.^5^

The clinical benefits of DDR are evident, producing dynamic image sequences similar to those obtained with fluoroscopy. The portable Konica Minolta system uses pulsed x‐rays to acquire sequential radiographs at 6 or 15 frames per second with pulse widths ranging from 5 to 16 ms. These images are compiled into a cine‐style video lasting up to 20 s. Across published studies of dynamic chest radiography (DCR), entrance skin doses range from approximately 0.4–4 mGy per acquisition at 15 fps and 4 ms pulse width with a 150 cm source‐to‐skin distance.^6^, ^7^, ^8^, ^9^, ^10^, ^11^ In comparison, the DDR system evaluated in this study uses longer pulse widths and generates higher radiation dose rates than conventional mobile fluoroscopy units. Although it delivers fluoroscopy‐like images, DDR is not classified as fluoroscopic equipment under current FDA regulations. The portable Konica Minolta DDR system is instead cleared as a mobile digital x‐ray system (21 CFR 892.1720, Class II) under FDA 510(k) number K221803.^12^ A key concern is that, unlike fluoroscopy systems, portable radiographic devices equipped with DDR lack a primary beam stop—a feature critical for radiation safety. While copper filtration is available as a dose‐reduction measure, the system's high output reinforces the potential need for shielding evaluations in rooms where DDR is used and the importance of ensuring appropriate personal protective equipment is worn throughout the procedure.

Currently, there is no published literature evaluating patient or personnel radiation exposure from portable DDR systems. This study aims to assess the radiation dose rates and shielding requirements for DDR and compare them to those of mobile C‐arm fluoroscopy systems commonly used in routine clinical practice.

## 2 METHODS

### 2.1 Imaging systems evaluated

Four mobile systems were evaluated: a Konica Minolta mKDR portable system (Konica Minolta, Tokyo, Japan) and three mobile C‐arms representing different vendors: a GE OEC Elite mobile C‐arm (GE Healthcare, Chicago, IL, USA), a Siemens Cios Spin mobile C‐arm (Siemens Healthineers, Erlangen, Germany), and a Ziehm Vision RFD 3D mobile C‐arm (Ziehm Imaging, Nuremberg, Germany). Comparison of the technical details regarding the systems are presented in Table 1. All four systems were used for primary beam characterization, whereas, only the DDR and GE C‐arm systems were used for scatter evaluation.

**TABLE 1:** Comparison of the technical specifications between the system used for this study.

Manufacturer	Konica Minolta	General Electric	Siemens	Ziehm
**Model name**	mKDR Xpress	OEC Elite CFD	Cios Spin	Vision RFD 3D
**FDA clearance (Year)**	2022	2017	2018	2017
**Tube potential (kV)**	40–150	40–120	40–125	40–120
**Tube current (mA)** ^a^	10–500	0.2–150	3–250	1.5–250
**Max operating data** ^b^	125 kV/320 mA	120 kV/20 mA; 20 mA/120 kV	125 kV/20 mA	120 kV/18 mA; 80 kV/20 mA
**Pulse rates (p/s)**	6, 15	4, 8, 15, 30	0.5–30	1, 2, 4, 8, 12.5, 25
**Pulse widths (ms)** ^c^	5–16	9, 19, 24, 29, 34	5–14	4–40
**Source‐detector distance (cm)**	Variable	100	116.4	106
**Anode angle (degrees)**	16	10	10	10
**Focal spot sizes (mm)**	0.7 and 1.3	0.3 and 0.6	0.3 and 0.5	0.3 and 0.6
**Total inherent filtration**	3.1 mmAl	6.75 mmAl at 75 kVp	6.95 mmAl at 75 kVp	4.3 mmAl + 0.1 mmCu
**Detector type**	Flat panel	Flat panel	Flat panel	Flat panel
**Detector conversion method**	Indirect	Indirect	Indirect	Indirect
**Scintillator material**	Cesium Iodide	Cesium Iodide	Cesium Iodide	Cesium Iodide
**Detector readout technology**	Thin film transistors (TFT)	Complementary metal‐oxide semiconductor (CMOS)	Complementary metal‐oxide semiconductor (CMOS)	Complementary metal‐oxide semiconductor (CMOS)
**Detector size (cm)**	36 × 43	31 × 31	30 × 30	31 × 31

^a^ Tube current is specified here as the lowest possible current to highest possible current observed in the system, including all imaging modes.

^b^ Maximum operating data are presented based on observations when the unit is operated in High‐Level Fluoroscopy (HLF) mode. The first line indicates the maximum tube current achievable at the highest tube potential, while the second line shows the maximum tube potential achievable at the highest tube current.

^c^ Pulse width values represent the range available across all imaging modes on the system.

### 2.2 Characterization of primary beam

**FIGURE 1:**
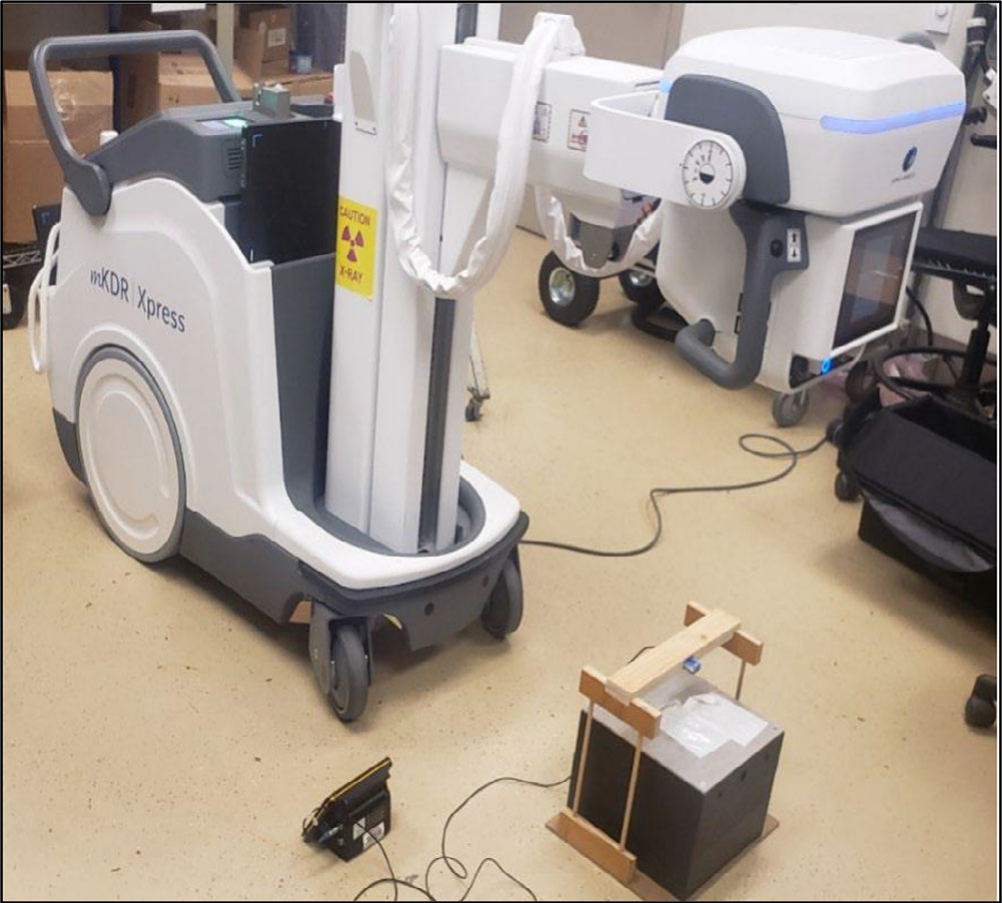
Setup of the dose‐rate measurement for the DDR technology. A source to image receptor distance (SID) of 100 cm, field size of 20 × 20 cm, and a 20 cm PMMA solid water phantom were used.

Unshielded primary air kerma measurements were performed at a standard source‐to‐image distance (SID) of 100 cm using a consistent setup across all four systems to ensure comparability (Figure 1). A RaySafe (Fluke Biomedical, Everett, WA) R/F solid‐state dosimeter positioned 1 m from the x‐ray source, was used for all measurements. For the DDR system, dose rates were assessed under three clinical dose settings: low, medium, and high. Additional data were collected for the DDR system at 6 and 15 pulses per second, with pulse widths of 5 and 16 ms. Mobile C‐arm measurements were acquired under three corresponding operational modes (Table 2), using the highest clinically available kV and mA combinations to represent the upper bound of dose output in routine practice. Pulse rate and pulse width for the C‐arm systems were chosen to closely match those used in the DDR system within clinical constraints. This protocol was designed to reflect realistic clinical use and allow for meaningful cross‐platform comparisons.

**TABLE 2:** Exposure parameters used to evaluate unshielded primary air kerma rates at 100 cm SID across all systems.

Manufacturer	Dose level	Tube energy (kVp)	Tube current (mA)	Pulse rate (p/s)	Pulse width (ms)
**DDR**	Low (1)	50	200	15	5
	Low (2)	50	200	6	16
	Medium (2)	80	500	15	5
	Medium (2)	80	500	6	16
	High (1)	125	320	15	5
	High (2)	125	320	6	16
**GE OEC Elite CFD**	Low	120	10	7.5	29
	Medium (Normal)	120	10	15	19
	High (Boost)	120	19	Continuous	–
**Siemens Cios Spin**	Low	120	10	7.5	13.3
	Medium (Normal)	120	10	15	10.6
	High (Boost)	125	19	30	8.3
**Ziehm Vision RFD 3D**	Low	120	4	8	23
	Medium (Normal)	120	8.8	12.5	23
	High (Boost)	120	62.5	25	10

*Note*: For each dose level, C‐arm settings reflect the highest clinically available kV and mA, while pulse rate and width were selected to closely match those used by the DDR system within clinical constraints.

### 2.3 Scatter radiation assessment

Unshielded secondary air kerma (scatter radiation) was evaluated for the DDR system. The methodology was adapted from Simpkin's framework, NCRP Report No. 147, and the study by Kai Yang et al.^13^, ^14^, ^15^, ^16^ Scatter‐to‐primary air kerma ratio per unit beam area and normalized scatter signal were measured using a RaySafe solid‐state detector, a Ludlum 9DP 230‐cc ion chamber, and a 20 cm^3^ solid water phantom.

The following summarizes the terminology and calculation methods used in scatter assessment. Since the scatter air kerma at 1 m from the phantom center (D_s_) is proportional to the primary air kerma at 1 m from the x‐ray tube focal spot (D_p_^1^), the scatter‐to‐primary ratio, a_sp_, is given by:

(1) $$a_{sp}=\frac{D_{s}}{D_{p}^{1}}\;$$

To account for the effect of field size, the scatter air kerma is assumed to vary linearly with the irradiated area, 𝐹, impinged on the phantom. This yields the scatter fraction, or the scatter‐to‐primary ratio per unit area, defined as:

(2) $$a_{f}=\frac{a_{sp}}{F}\;$$

A scatter fraction curve for the GE C‐arm was also generated using its manufacturer‐provided isokerma map, normalized to the same measurement setup and exposure parameters as used for the DDR system.^17^ Although three mobile C‐arm systems were evaluated for primary dose measurements, only the GE OEC Elite system was included in the scatter analysis, as its performance is representative of modern mobile C‐arm units. Scatter distributions among systems with similar geometry, filtration, and imaging parameters are generally comparable. Thus, including additional systems was not expected to provide substantially different results or affect the comparative conclusions regarding DDR.

To evaluate the normalized scatter signal, exposure rate measurements were divided by the total mAs delivered per exposure. The total mAs was calculated as the product of pulse width, tube current, and pulse rate. The normalized scatter was therefore computed using:

(3) $$\begin{array}{c} \mathrm{Normalized}\;\mathrm{scatter}\;\;\left[\frac{\mathrm{mGy}}{\mathrm{mAs}}\right]\\ \;=\frac{\mathrm{Exposure}\;\mathrm{Rate}\;\left[\frac{\mathrm{mGy}}{s}\right]}{\mathrm{Pulse}\;\mathrm{width}\;\left[s\right]*\mathrm{Tube}\;\mathrm{Current}\;\left[\mathrm{mA}\right]*\mathrm{Pulse}\;\mathrm{Rate}\;\left[\frac{1}{s}\right]} \end{array}$$

(4) $$\mathrm{Normalized}\;\mathrm{scatter}\;\;\left[\frac{\mathrm{mGy}}{\mathrm{mAs}}\right]=\frac{\mathrm{Exposure}\;\mathrm{Rate}\;\left[\frac{\mathrm{mGy}}{s}\right]}{\mathrm{Total}\;\mathrm{mAs}\;\left[\frac{\mathrm{mAs}}{s}\right]}$$

**FIGURE 2:**
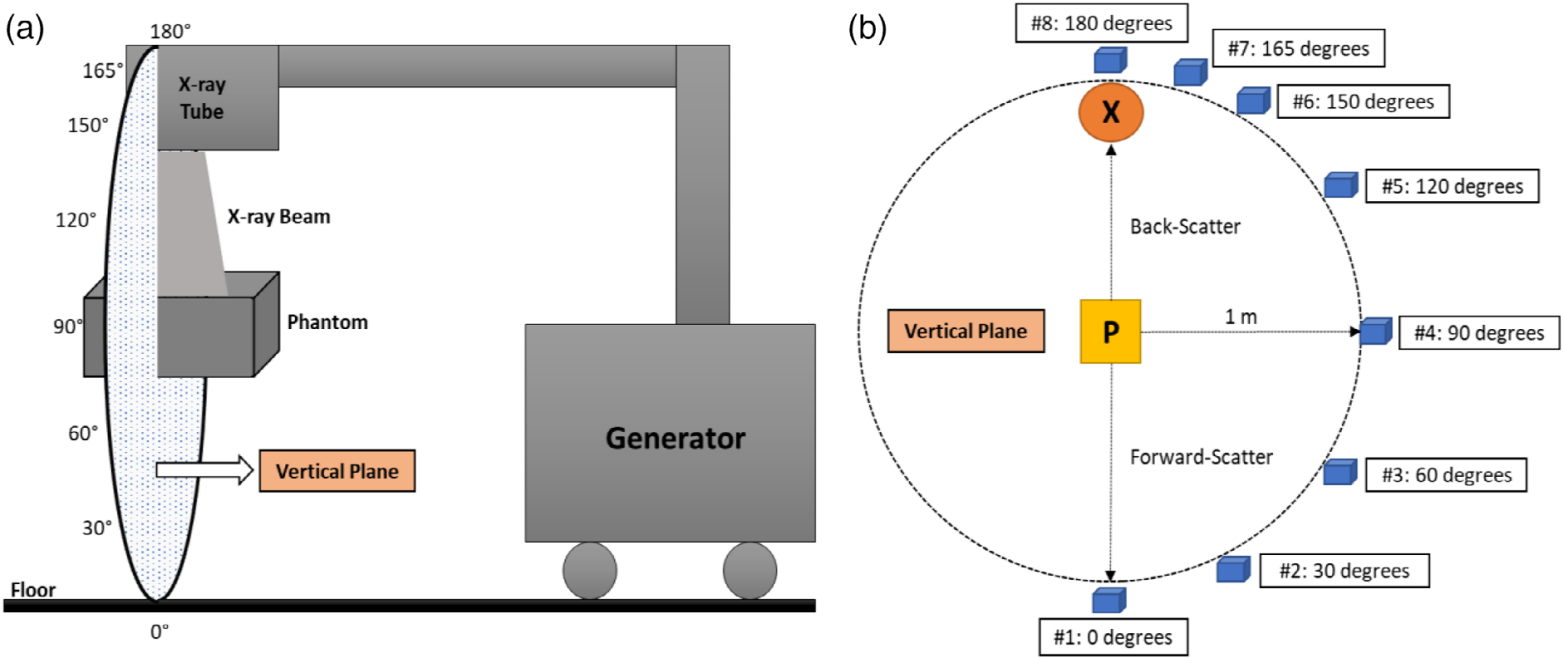
(a) Illustration of the vertical plane used in the experimental setup for scatter radiation measurements at eight discrete angles, each positioned 1 meter from the center of the phantom (not drawn to scale). (b) 2D schematic of the experimental setup. The ion chamber is represented by cubic boxes, the x‐ray tube by a circle labeled “X,” and the phantom by a central rectangle labeled “P.” Measurements at each angle were performed using five different tube potential settings.

The experimental setup was based on a modified version of the geometry described by Yang et al. To ensure consistency with the assumptions used in the shielding assessment, all measurements were acquired with the image receptor removed from the beam path. This configuration reflects a worst‐case scenario in which the primary beam is unintentionally directed toward a barrier without attenuation from the detector. Scatter measurements were performed in a single plane at eight discrete angles using a 20 cm^3^ solid water phantom positioned 1 meter from the focal spot. The incident field size on the phantom was set to 400 cm^2^. The ion chamber was placed 1 meter from the phantom center at each angular position for five tube potential settings (60, 75, 90, 110, and 125 kV). Because the ion chamber was calibrated with a Cs‐137 source, all exposure rate readings were corrected using the energy response curve provided in the manufacturer's datasheet, which accounts for the detector's relative response at the x‐ray energies used in this study. Figure 2 illustrates the angular layout of the measurement setup.

In addition to evaluating the scatter fraction and normalized scatter, we generated the differential cross section for Compton scattering of photons by electrons at those five tube potential settings, focusing specifically on scattering angles between 90° and 180°. The Klein–Nishina formula describes the probability of a photon scattering into a given angle after interacting with an electron.^18^ This relationship is expressed in Equation (5), where a photon with initial energy, 𝐸, is scattered into angle, θ, with resulting energy, 𝐸′:

(5) $$\frac{d\Omega }{d\sigma }=\frac{r_{o}^{2}}{2}\;{\left(\frac{E^{\prime }}{E}\right)}^{2}\left(\frac{E^{\prime }}{E}+\frac{E}{E^{\prime }}-\sin^{2}\theta \right)$$

where,

$\frac{d\Omega }{d\sigma }$: Differential cross section (in units of $\frac{m^{2}}{\mathrm{sr}}$)

*r*_o_: Classical electron radius

*E*: Incoming photon energy

*E’*: Scattered (outgoing) photon energy

Θ: Scattering angle (angle between incident and scattered photon)

The relationship between the incident and scattered photon energies is given by the Compton formula:

(6) $$E^{\prime }=\frac{E}{1+\frac{E}{m_{e}c^{2}}\left(1-\cos\theta \right)}\;$$

All calculations and plots of the differential cross section as a function of angle were performed using Python 3.12 (Python Software Foundation, www.python.org).

**FIGURE 3:**
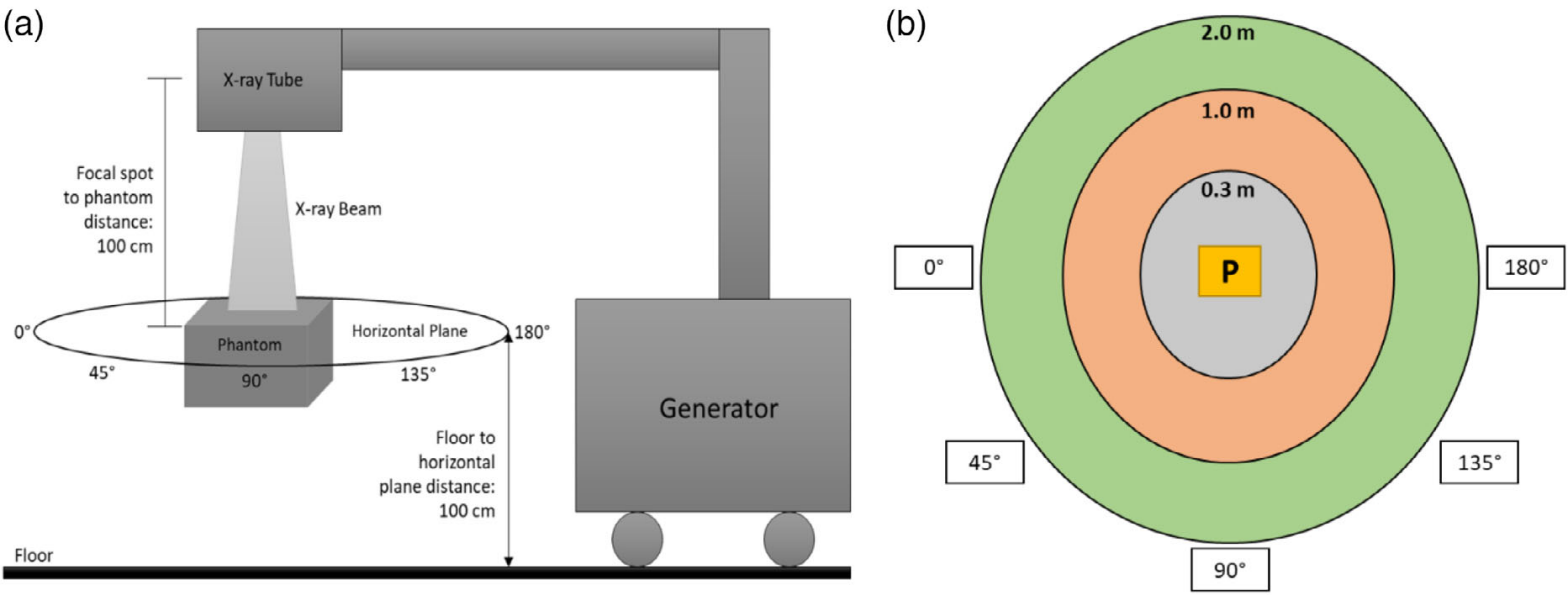
(a) Side‐view schematic of the DDR system illustrating the experimental setup used for scatter measurements. The x‐ray tube directs the beam toward a phantom positioned below, with the generator shown to the right of the setup.(b) Top‐down schematic representation of the horizontal measurement plane intersecting the center of the phantom, labeled “P”. Scatter was measured at three radial distances (0.3, 1.0, and 2.0 m) and five angular positions. In this coordinate system, 0° corresponds to the front of the system (away from the generator), 180° toward the generator, and 90° represents the lateral directions.

To complement the theoretical analysis and enable comparison between systems, additional scatter dose measurements were performed during a 20‐s exposure using both the DDR and GE C‐arm systems. The phantom was positioned 100 cm from the focal spot and elevated to a height of 100 cm from the floor. Scatter was recorded at five angular positions along a horizontal plane at distances of 0.3, 1.0, and 2.0 m from the phantom center. Both systems were operated at their highest dose settings; for the DDR, a pulse rate of 6 pulses per second and a 16‐ms pulse width were selected. A Radcal survey meter was used to acquire all measurements. These measurements provide an estimate of the scatter dose that personnel may receive to the waist or chest region during a 20‐s exposure at typical working distances for these systems. A layout of this measurement setup is provided in Figure 3.

### 2.4 Leakage radiation and image receptor attenuation analysis

Leakage radiation was measured at three positions around the x‐ray tube: left, right, and back. To ensure complete attenuation of the primary beam, the collimators were fully closed and a 3.6 mm lead sheet was placed over the tube opening. The ion chamber was positioned 1 meter from the focal spot for each measurement. The system was operated at its maximum output setting: 125 kV, 320 mA, 6 pulses per second, and a 16 ms pulse width.

Data on image receptor attenuation of the primary beam were also collected. The R/F dosimeter was positioned at 100 cm, and primary air kerma measurements were taken with and without the image receptor in the beam. The beam's attenuation factor was calculated based on the general attenuation equation Equation (7).

(7) $$I=I_{0}*e^{-\mu x}$$

### 2.5 Shielding requirements estimation

Shielding requirements for the DDR system were estimated using NCRP Report 147 guidelines to determine appropriate protective measures and evaluate the shielding thickness required for the high‐dose setting. The barrier transmission factor (B) was calculated using Equation (8) to assess shielding effectiveness.

(8) $$B=\left(\frac{P}{T}\right)\;*\left(\frac{d^{2}}{K^{1}*U*N}\right)$$

where,

*P* is the shielding design goal.

 *d* is the distance to the occupied area.

*K* ^1^ is the dose per patient at 1 m distance.

*T* is the occupancy factor.

*U* is the use factor of the x‐ray beam.

*N* is the number of patients per week.

The required concrete thicknesses for a patient room using the DDR system were then determined based on Equation (9) and the fitting parameters (α, β, γ) were obtained from NCRP 147 Table B.1 and C.1.^15^ These tables provide empirical transmission parameters for standard shielding materials such as concrete and are specific to primary radiation (Table B.1) and secondary radiation (Table C.1). It is important to note that the shielding calculations in this study assume standard‐weight concrete. Various combinations of patient volume, occupancy factors, and distances were applied to evaluate how these factors affect the required thicknesses and to understand the potential changes in thickness requirements if any of these parameters were altered.

(9) $$x_{\mathrm{barrier}}=\frac{1}{\alpha \gamma }\;*\ln\left(\frac{{\left(\frac{1}{B}\right)}^{\gamma }+\frac{\beta }{\alpha }}{1+\frac{\beta }{\alpha }}\right)$$

## 3 RESULTS

### 3.1 Primary dose rate comparison

**FIGURE 4:**
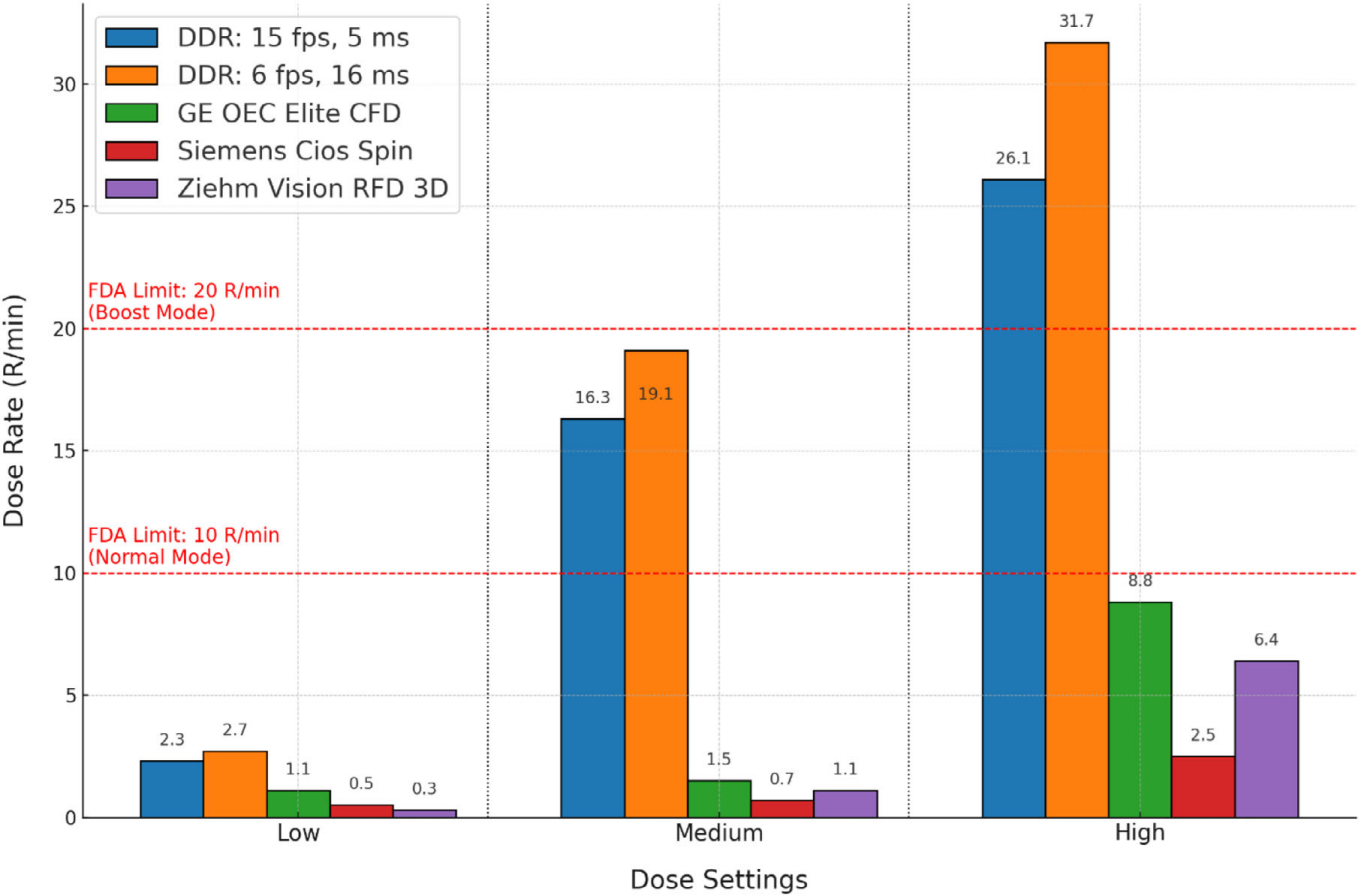
Comparison of primary exposure rates between DDR and commonly used C‐arm systems (GE OEC Elite CFD, Siemens Cios Spin, and Ziehm Vision RFD 3D) across low, medium, and high dose settings. DDR demonstrates higher dose rates with longer pulse widths and exceeds C‐arm output at medium and high dose settings, consistent with its high‐fluence design for dynamic imaging. FDA patient entrance air kerma rate limits are shown for reference.

The dose rate measurements (Figure 4) show a consistent increase in exposure with longer pulse widths (16 ms at 6 p/s) compared to shorter pulse widths (5 ms at 15 p/s) for the DDR system. This trend is evident across all dose settings and becomes more pronounced at higher tube currents and energies, highlighting the effect of pulse duration on cumulative dose. It reflects a fundamental trade‐off in dynamic imaging: longer pulse widths at lower pulse rates increase dose efficiency but reduce temporal resolution, whereas shorter pulse duration improves temporal fidelity at the cost of higher dose.

Comparisons between DDR and C‐arm measurements at the same SID shows a substantial difference in radiation output. At low‐dose configurations, DDR and C‐arm systems deliver comparable dose rates. However, as dose settings increase, DDR systems consistently demonstrate significantly higher output—particularly at medium and high dose settings—where DDR dose rates exceed those of C‐arms by factors of two to three. This disparity highlights the DDR system's design philosophy, which prioritizes high‐resolution, high‐speed dynamic acquisition, potentially best suited for applications such as chest or orthopedic motion studies. Such performance necessitates higher photon fluence and explains the use of tube currents as high as 500 mA in DDR systems, compared to sub‐20 tube currents in conventional C‐arms.

### 3.2 Angular and energy‐dependent scatter analysis

**FIGURE 5:**
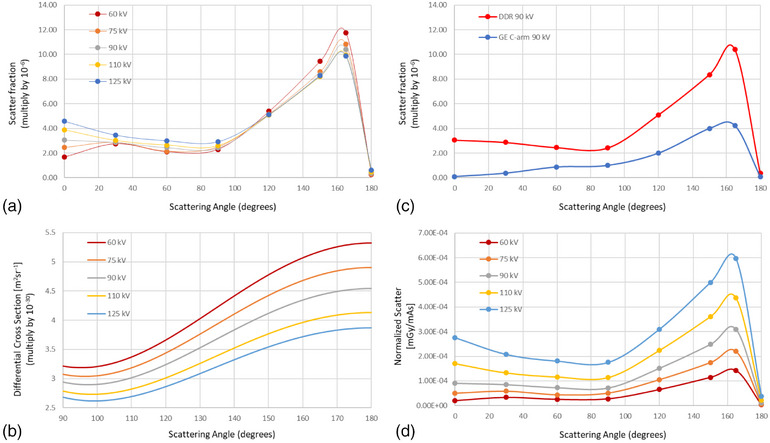
Scatter radiation distribution between 0° and 180°. (a) Scatter fraction per cm^2 field size at 1 m primary distance. (b) Differential cross section for Compton scattering of photons as a function of scattering angle (90°–180°) for five x‐ray tube potentials: 60, 75, 90, 110, and 125 kV. (c) Comparative analysis of scatter fraction between DDR and C‐arm system at 90 kV. (d) Normalized scatter signal (mGy/mAs) measured at 1 m away from the phantom center for various tube energies used clinically.

Figure 5 presents the results of the angular scatter distribution measurements. Due to the absence of the image receptor in the primary beam path, an elevated forward scatter is observed at 0°, as shown in Figure 5a, where the receptor would typically provide attenuation. Both the scatter fraction and normalized scatter signal exhibit a clear peak at 165°, indicating strong backscatter characteristics for the DDR system. A sharp decline is observed near 180°, mainly due to attenuation by the x‐ray tube housing. The shape of the measured scatter fraction curve closely resembles that derived in NCRP Report No. 147 for a tungsten anode and aluminum filtration. However, a notable divergence appears beyond 90°, where lower‐kV beams exhibit higher scatter fractions than higher‐kV beams. This results from a combination of differences in total system filtration, anode angle, and energy‐dependent Compton scattering probabilities. As shown in the differential cross section for photon–electron scattering (Figure 5b), the probability of backscatter decreases with increasing photon energy, which helps explain the observed reduction in backscatter for higher‐kV beams beyond 90°.

These trends are also reflected in the comparative analysis between the DDR and GE C‐arm systems (Figure 5c). Across all angles, the DDR system consistently demonstrates higher scatter fractions, which can largely be attributed to differences in total inherent filtration. As detailed in Table 1, the DDR system has 3.1 mmAl filtration compared to 6.75 mmAl in the GE C‐arm.

Furthermore, the normalized scatter signal plot (Figure 5d) supports that the DDR system produces the highest scatter at 165° relative to the primary beam and phantom center. The trend is consistent with theoretical expectations: higher beam energies result in greater overall scatter and more forward‐directed scatter. For all beam energies evaluated, the least amount of scatter was observed at 60° and 90°.

**FIGURE 6:**
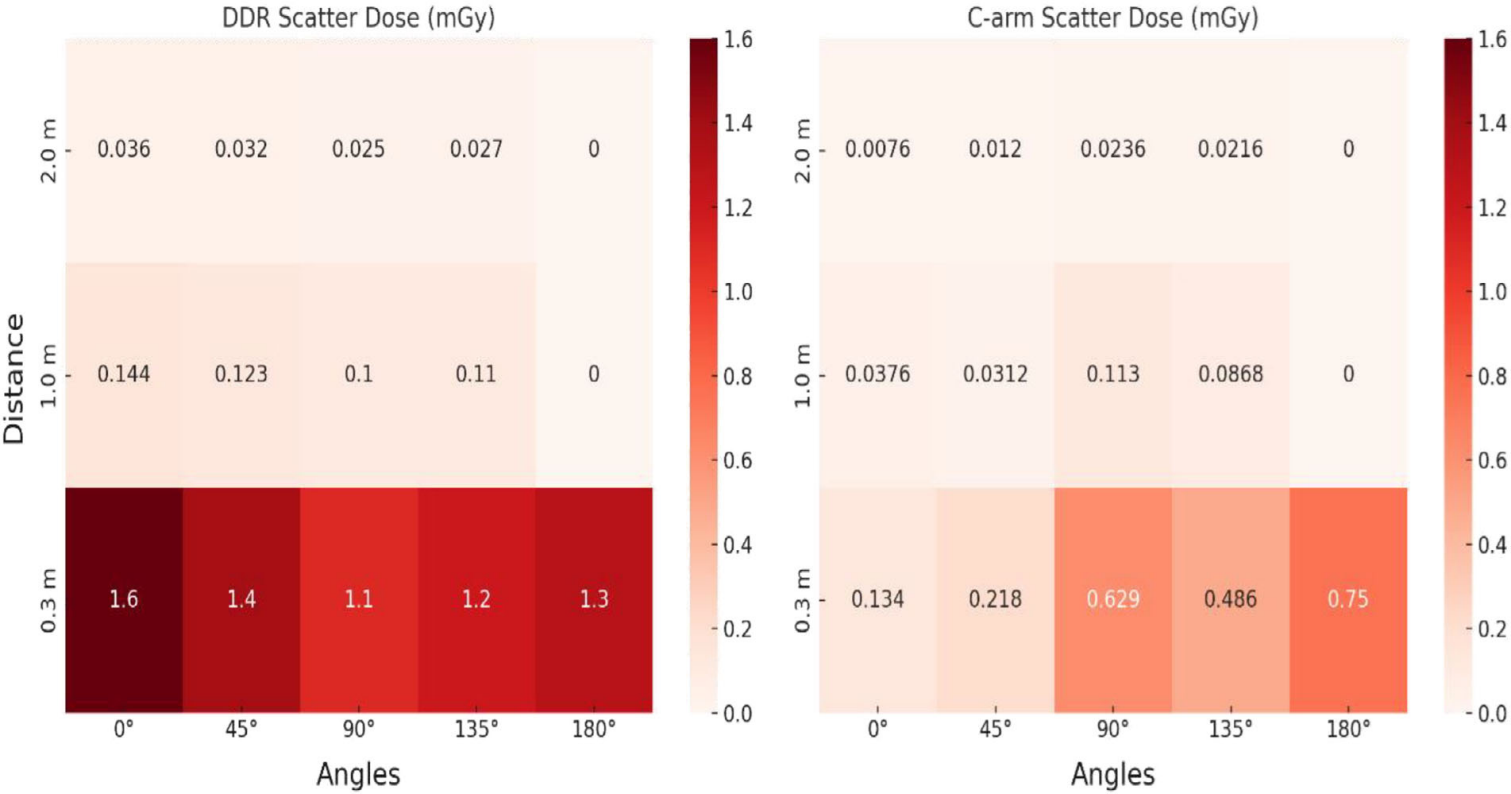
Comparison of scatter dose measurements for DDR and C‐arm across multiple angles and distances. DDR exhibits higher scatter doses at most positions, with the difference being most pronounced at closer distances. Scatter doses decrease with increasing distance for both systems, though DDR maintains measurable exposure even at 2.0 m.

Figure 6 shows the scatter dose comparison between the DDR and GE C‐arm systems across multiple angles and distances in the horizontal plane at a height of 100 cm above the floor. The DDR system consistently produced higher scatter doses than the C‐arm across nearly all measured positions. At 90° and beyond, scatter doses at 1.0 and 2.0 m were generally similar between systems, with a comparable trend also observed at 45° for the 2.0 m position. No measurable scatter was observed at 180° for either system at 1.0 or 2.0 m due to attenuation from the system housing and generator. For the DDR system, the highest scatter dose would be received by personnel standing directly in front of the unit (0°), while for the C‐arm system, it appeared to occur in the opposite direction (180°).

### 3.3 Leakage radiation and beam attenuation

Leakage radiation was highest on the left side of the x‐ray tube (cathode side) compared to the right (anode side) and back. In all cases, leakage signals were two to three orders of magnitude lower than the scatter signals reported in Figure 5d and remained well below the FDA regulatory limit of 100 mR/h at 1.0 m distance. The measured leakage values were 1.69E‐06 mGy/mAs (left), 8.37E‐07 mGy/mAs (right), and 9.34E‐07 mGy/mAs (back).

In the evaluation of the image receptor attenuation factor, it was found that the receptor attenuated approximately 98% of the beam when positioned in the path of the primary x‐ray. This emphasizes the importance of proper field alignment with the image receptor to minimize unnecessary radiation exposure.

### 3.4 Shielding thickness requirements

For the shielding evaluation, several combinations of expected workload, occupancy, and weekly patient volume were considered, based on anticipated acquisition parameters for different procedures. Table 3 presents the results for a worst‐case scenario, defined as shielding calculations performed at the highest output parameters. In this case, the workload was based on the total mAs per exposure, assuming a full 20‐s exposure duration. Barrier distances of 4.1 m for primary and 3.0 m for secondary shielding were applied, in accordance with NCRP Report No. 147. Secondary radiation included both scatter and leakage components. The maximum measured scatter value, occurring at 165° as shown in the normalized scatter plot, was used in the calculation, with both scatter and leakage assumed to be directed perpendicularly to the shielding surface.

**TABLE 3:** Shielding requirements for the DDR system based on patient load, occupancy factor, and distance to occupied areas.

**Primary air kerma at 1** **m (mGy/patient)**	92.65			
**Secondary air kerma at 1** **m (mGy/patient)**	0.37			
**Max scattering angle (degree)**	165			
**Max scatter fraction (cm^−2^)**	9.89E‐06			
**Field size (cm^2^)**	400			
**Patient volume (patients/week)**	5		15	
**Total workload (mAs/week)**	3072		9216	
**Max scatter at 1** **m (mGy/week)**	1.83		5.50	
**Occupancy (T)**	0.025	1	0.025	1
**Primary concrete thickness (mm)**	37.55	115.93	57.40	143.83
**Secondary concrete thickness (mm)**	–	21.29	–	38.24

*Note*: Worst‐case scenario results are presented in this table. The following acquisition parameters were used: 125 kV, 320 mA, 6 pulses per second, 16 ms pulse width, 20 s exposure.

As shown in Table 3, shielding requirements are strongly influenced by both weekly patient volume and occupancy of adjacent areas. Concrete thickness nearly triples when moving from low‐ to high‐occupancy conditions. While occupancy has the most significant impact, increasing the number of procedures per week also leads to a moderate but measurable increase in shielding demand. Additionally, the required concrete thickness is largely determined by the shielding needed for the primary beam, indicating that secondary radiation, scatter and leakage, makes only a minimal contribution to the overall shielding requirements. This highlights a general trend that, under typical DDR operating conditions, shielding design is predominantly driven by the primary radiation workload.

## 4 DISCUSSION

This study provides a comprehensive characterization of radiation dose and scatter output from portable DDR system, benchmarking it against common mobile C‐arm systems. While DDR enables dynamic imaging on a portable platform, the findings highlight several safety and operational considerations that are essential for its responsible clinical use.

Our measurements confirm that DDR operates at much higher dose rates than traditional mobile C‐arms, particularly under medium and high exposure settings. These elevated dose outputs approach or exceed FDA limits for conventional fluoroscopy systems.^19^ Although DDR is not classified as fluoroscopy, its radiation profile behaves similarly and, in some cases, surpasses fluoroscopy in terms of exposure. This highlights the need for careful parameter selection. DDR may offer unique diagnostic value in dynamic pulmonary or orthopedic studies, but its use should be judicious, with imaging protocols tailored to minimize dose without compromising diagnostic quality. While this study did not directly evaluate diagnostic performance, the higher output of DDR is anticipated to improve visualization of motion‐dependent anatomy and function. Further clinical studies will be necessary to quantify image quality gains and diagnostic benefit.

Another important distinction is the absence of a primary beam stop in DDR systems. As a result, precise beam alignment with the image receptor is critical. Misalignment can result in unattenuated primary radiation reaching occupied areas, including adjacent rooms or lower floors. Shielding analysis revealed that both workload and occupancy factor play critical roles in determining barrier thickness requirements. While typical hospital floor thickness ranges from 140–280 mm and wall thickness around 150 mm, required shielding for DDR may exceed these values under high workload or full‐occupancy conditions.^20^ Based on the authors’ institutional experience across buildings of varying construction vintages, actual concrete thickness has been found to range from as little as 50 mm of standard‐weight concrete to as much as 180 mm of lightweight concrete. In the worst‐case scenario modeled in this study, 15 patients per week in a fully occupied area, shielding requirements reached up to 145 mm of standard‐weight concrete. Particularly, even small reductions in thickness can lead to large increases in transmission, reinforcing the need for conservative shielding plans. To mitigate risk, portable DDR systems should ideally be used in areas adjacent to low‐occupancy zones and rooms with known structural adequacy.

The study also demonstrates that DDR produces higher scatter radiation than C‐arm systems at most angles and distances. The use of high tube currents and relatively low filtration contributes to this increased scatter, which presents an occupational exposure risk to personnel, especially when standing within 1–2 m of the patient. Staff should maintain as much distance as possible during image acquisition, ideally at least 2 m, and use personal protective equipment including lead aprons and leaded eyewear. When proximity is unavoidable, such as during repositioning or device checks, exposure should be minimized through timing and shielding techniques. In addition to these protective measures, system‐level dose‐reduction strategies can be employed. These include the use of additional copper filtration (e.g., 0.1–0.2 mm Cu) to lower entrance skin dose, tight collimation to reduce the irradiated field and scatter, and minimizing frame rate, pulse width, or total acquisition time.^21^, ^22^, ^23^, ^24^, ^25^

While the study used well‐controlled measurements and realistic clinical parameters, several limitations must be acknowledged. The use of a homogeneous PMMA phantom does not fully replicate patient‐specific anatomy or tissue composition. In thoracic imaging, for example, variable lung density and internal structures can result in more forward‐directed scatter and a broader angular distribution than what was measured in this study.^26^ System comparisons were limited by differences in technical design and dose delivery constraints. Additionally, shielding estimates assume worst‐case usage and may not reflect daily practice in lower‐volume sites. Another limitation is the absence of real‐world occupational exposure data from clinical DDR use, as only one case had been performed at our institution during this study period. As DDR adoption increases, future work should include personnel dosimetry during actual procedures to validate and refine the estimates presented here.

## 5 CONCLUSION

DDR systems provide portable dynamic imaging capabilities but deliver substantially higher radiation output than conventional mobile C‐arms. In addition, scatter dose rates from DDR were approximately 1.5–3 times higher than those from a conventional mobile C‐arm under comparable conditions. This elevated dose, driven by high tube currents and long pulse durations, raises important safety concerns for patients, personnel, and shielding infrastructure. While DDR offers potential clinical value in motion‐sensitive applications, its safe integration into practice requires careful protocol selection, attention to scatter exposure, and thoughtful shielding planning of exam rooms where the system will be used. As DDR systems become more prevalent and approach fluoroscopic performance, regulatory and design guidance may need to evolve to reflect their unique operational profile.

## AUTHOR CONTRIBUTIONS

**Azmul Siddique**: conceptualization; methodology; data curation; formal analysis; investigation; visualization; writing—original draft; writing—review & editing. **Gary Ge**: supervision; writing—review & editing. **Jie Zhang**: supervision; writing—review & editing. All the authors have read and approved the final version of the manuscript.

## CONFLICT OF INTEREST STATEMENT

The authors declare no conflicts of interest.

## Data Availability

Authors will share data upon request to the corresponding author.
